# The role of agriculture in women’s nutrition: Empirical evidence from India

**DOI:** 10.1371/journal.pone.0201115

**Published:** 2018-08-15

**Authors:** Tanvi Rao, Prabhu Pingali

**Affiliations:** 1 IMPAQ International LLC, Washington, D.C., United States of America; 2 Charles H. Dyson School of Applied Economics and Management, Cornell University, Ithaca, New York, United States of America; SOAS, University of London, UNITED KINGDOM

## Abstract

In this paper, we establish a statistically important relationship between household agricultural income and women’s BMI using a five-year panel dataset of rural households drawn from 18 villages across five Indian states. Using within household variation over time, we estimate both the extent to which short-term changes in agricultural income are associated with short-term changes in BMI, and the effect of agricultural income growth on BMI growth over a longer term. Over the longer term, and for the group of households that regularly farm, we find a 10 percentage point agricultural income growth to be associated with a 0.10 percentage point growth in BMI. Consistent with the literature, this effect is economically modest, but important considering that we do not find a corresponding effect for growth in non-agricultural income. We show that both the own-production and market purchase of food are associated with nutritional improvements. While women’s BMI is associated with an increase in the consumption of own-produced cereals, the market plays an important role in facilitating access to more nutritious foods like pulses. Lastly, we also find that effects of agricultural income are driven by younger women, in the age-group 15-25 years, who face a particularly strong nutritional disadvantage in India.

## Introduction

Low maternal body-mass index (BMI), generally defined as a BMI of less than 18.5 kg/m^2^, is a grave public health concern because its implications extend well beyond the individual herself. Maternal undernutrition contributes to fetal growth restriction, which increases the risk of neonatal deaths and, for surviving children, of stunting [1]. Indian women are particularly at risk of being too thin and it is estimated that approximately 42.2% of pre-pregnant women in India are underweight [2]. In yet another stark manifestation of the “Asian Enigma” [3], in Sub-Saharan Africa, only 16.5% of pre-pregnant women are estimated to be underweight, even though they are much poorer.

Among the reasons advanced for the poor nutritional status of Indian women, an enduring explanation relates to the intra-household status of women. Several indicators of women’s status in the literature consistently rank women in the countries of South Asia as lower in comparison to their counterparts in Asia, Africa, Latin America and the Caribbean [4]. The Indian case is particularly unique whereby features of familial structure and cultural norms that designate inter-personal hierarchies foster low social-status among women with perpetuating consequences for her own and her child’s health [5]. Worryingly, recent numbers emerging from the Rapid Survey on Children (RSOC) conducted by the Union Ministry of Women and Child Development and UNICEF show that while India has seen encouraging progress on metrics of child malnutrition since 2005, the situation for adolescent girls aged 15-19 years has barely budged with close to 45% of girls in the age-group having BMI of less than 18.5. With this context in mind, policy interventions that have the potential to increase the bargaining power of women hold particular promise in addressing the problem of maternal malnutrition in the country.

Recent academic and policy interest in leveraging the agricultural sector in developing countries to combat the scourge of malnutrition is motivated, in part, by the fact that agriculture is not only a major employer overall in these countries, but is a major employer of women in particular [6], [7], [8]. Therefore, one important pathway by which agriculture is linked to nutrition is by way of being a source of income for women, which in turn can influence the intra-household allocation of food and other nutrition-enhancing complements. This hypothesis follows from a large body of empirical literature which finds that the identity of the income-earner matters in determining the distribution of family expenditures among different uses and women, more so than men, have been found to devote earnings to collective household consumption needs [9], [10], [11]. At the same time, heavy agricultural workloads and exposure to toxins and disease through agricultural activities can deleteriously affect women’s health and nutrition and also have negative consequences for lactation and child-care [12], [13]. Therefore, the net implications of increases in agricultural income, for women’s nutrition require empirical investigation. Other pathways by which agriculture and nutrition are posited to be linked include, production for own-consumption (particularly relevant in the face of high transaction costs and missing markets for nutritious foods), overall income effects for net-sellers of food and price-effects for net-buyers [14], [15].

In a narrative synthesis of the existing malnutrition literature in India authors in [16] find less than ten papers in peer-reviewed journals that empirically examine different determinants of women’s nutrition, as measured by anthropometric outcomes (also see [14] for a related and relevant review). Moreover, all of these studies use cross-sectional data, and most of them do not extensively control for confounding effects. One constraint is the availability of anthropometric data, which is strikingly lacking. Periodic National Family and Health Surveys (NFHS) which collect nationally representative anthropometric data on children and adults do not collect detailed income and agriculture data and are collected infrequently. For instance, the latest round of NFHS data was released in January 2018, around a decade after the release of the previous round. In this paper we respond to this gap by using five years of household-level panel data, from 18 villages across 5 Indian states, collected by the International Crops Research Institute for the Semi-Arid Tropics (ICRISAT) as part of the Village Dynamics in South Asia (VDSA) program, to establish the link between household agricultural income and women’s nutritional status, and explore the pathways by which agricultural incomes may affect nutrition.

Some existing findings, on agricultural output and malnutrition, in the literature provide context and aid in the interpretation of our results. The effect of agricultural production on malnutrition at the state-level is inconclusive in the Indian context. For instance, [17] find a modest effect of state-level agricultural production on both child and adult malnutrition metrics but use cross-sectional data with few controls. On the other hand, [18], finds the effect of state level agricultural growth on childhood stunting, with state fixed effects, to be particularly weak for Indian states. In a household fixed-effects study, using Tanzanian data, [19], establishes a statistically important, positive (inelastic) effect of household harvest value of crops on height-for-age z-scores of children under 5. However, the author does not find any effects of the same for adults. This could occur because adult underweight is not a substantial problem in their context, with only about 9 percent of the adult sample being underweight. Using methods comparable to ours and of [19], authors in [20] use three years of data to examine effects of sector-specific incomes in Uganda. They do not find agricultural incomes to play a crucial role in improving measures of child malnutrition, but caution that their results are heavily context specific to the agricultural and dietary profile of Uganda.

Some recent studies also throw light on specific pathways by which agriculture may affect nutrition. For instance, [21], finds that there is a link between household-level production diversity and diversity of diets among pre-school children in rural Ethiopia, but it breaks down for households that have market access to food. The potential of two agricultural pathways, production for own-consumption (measured by production diversity) and income effects (measured by agricultural revenue), on household dietary diversity has been looked at in the Nigerian context [22]. The authors find both pathways to have statistically significant but relatively inelastic effects on household dietary diversity. Our findings strengthen the existing literature in multiple ways. Firstly, we show that controlling for agricultural sector participation, there is a statistically significant relationship between household agricultural income and individual nutrition. We do so both by utilizing within household, year to year, variation in household agricultural income and by associating growth in agricultural income with growth in BMI over the longer term. Here, we contribute to the limited pool of estimates, across countries, which provide a measure for the agricultural income elasticity of anthropometrics. Further, we test for whether BMI increases among women are associated with production for own consumption or market purchase of food, for different food groups. We document an interesting pattern whereby the own production of cereals is positively associated with increased BMI on the one hand, and on the other hand, market purchase of pulses is positively correlated with BMI increases. This suggests that the production for own consumption pathway is beneficial for nutrition only in the case of cereals, though maternal nutrition may benefit from market purchase of more expensive and nutritious foods like pulses. Finally, we establish heterogeneous effects by women’s age and show a significantly higher impact of agricultural income on the nutritional status of younger women vis-a-vis older women.

## Data & summary statistics

This paper uses five years (2009-2013) of publicly available household and individual level panel data collected by ICRISAT as part of the VDSA program. The data are drawn from 18 villages across 5 Indian states Andhra Pradesh/Telangana, Gujarat, Karnataka, Maharashtra and Madhya Pradesh. The total number of individuals with valid BMI data in the sample varies from year to year; and ranges between 791-992 individuals. Rainfall data are from the University of Delaware Air Temperature and Precipitation database.

S1 Table lists the key variables we use in our analysis along with their means and standard deviations. Anthropometric data are collected annually, at the beginning of the survey cycle, and income and consumption data are collected monthly, in subsequent months. In view of this feature of the survey design, we lag all our explanatory variable by a year, to predict the following year’s BMI, our outcome variable of interest. Thus, BMI data used in the study applies to years 2010-2013 (anthropometric data collection in VDSA villages, from the five states, started in 2010) and data from 2009-2012 are used for the explanatory variables. This ensures that BMI data in every year is measured after income and expenditure data for the year. S1 Fig plots the cross-sectional distribution of the BMI of sample women. BMI data that are likely measured with error are excluded from the analysis. For all individuals having BMI less than 11 and greater than 40, and for individuals with individual-level BMI deviations smaller than the 1st percentile and larger than the 99th percentile, we consider observations of the individual for all years, on a case-by-case basis, to classify the BMI observation as measurement error or not. This is done, primarily, based on consistency of height values recorded in other years for the individual. In all, only 0.42% of all BMI observations are excluded on account of being measurement error. Among all women of child-bearing age, between 15-49 years, 33% of women are underweight with BMI<18.5. This number is worryingly high, and shows little progress since the DHS national average of 35% underweight women in 2005. Moreover, the incidence of underweight for younger women (15-25 y), among whom fertility is primarily concentrated, is higher by more than 13 percentage points, with the BMI distribution for this age-group having a larger mass of observations in the low BMI ranges. While on the other extreme, problems of overweight and obesity are also prevalent in India, in our sample the incidence of obesity is relatively small–roughly 1.76% of women are obese with BMI>29.9 kg/m^2^.

S2 Fig plots the distribution of the deviations of women’s year-specific BMI from their mean BMI. As is to be expected, within-individual variation in BMI, which we will be utilizing for our panel data analysis is more limited, but nevertheless sufficient to yield meaningful insights. The average yearly deviation from individual-specific BMI means is 0.65 points and for 95 percent of the women in our sample BMI varies within a band of +/- 2 BMI points. Even though we explicitly check for and exclude BMI observations that are undoubtedly measured with error (primarily based on older individuals for whom a lower height was recorded in a subsequent year), there are still some observations with large absolute deviations in BMI, owing to wide fluctuations in the weight of the individual over the span of four-years. We examine how our results are affected by the presence of such outlier individuals in subsequent analysis.

Another factor that may contribute to measurement error of the BMI variable is that some women could be pregnant during the years of the study. Even though the ICRISAT dataset does not explicitly report pregnancy status, we construct a binary variable to denote the years in which a woman is pregnant based on the birth-date of her children, which is backed out using child age and date of interview. We estimate our main results by accounting for this source of measurement error and report findings in the results section. Because we back out pregnancy status based on child age, we are only partially able to observe pregnancy status for 2013 which is the final year in our study sample. For this year, while we observe women who were pregnant in 2013 and gave birth in 2013, we cannot observe those women who are pregnant in 2013 but give birth in 2014. Therefore, while our results are both robust to and improve in precision upon excluding pregnant woman observations, a word of caution applies.

## Empirical specification

We set-up our econometric framework to (a) quantify the effect of agricultural income on women’s BMI over the short-term by modeling year-wise deviations in BMI from person-specific means as a function of deviations in household agricultural income; (b) estimate the growth in women’s BMI, over a span of four years, as a function of the growth in her household’s agricultural income. The growth specification averages out positive and negative yearly fluctuations in income and helps to estimate the cumulative effect of income from agriculture over time. We examine the effect of agricultural income on individual nutritional status over the short-term (one year) using the regression specification in Eq (1):

$$BMI_{ihvt}=\alpha_{ihv}+\beta_{1}f\left(AgInc_{hvt-1}\right)+\beta_{2}A_{hvt-1}+\beta_{3}P_{hvt-1}+\beta_{4}X_{hvt-1}+\gamma_{ih}+\varepsilon_{ihvt}$$ (1)

As an indicator of individual nutritional status, we are interested in the BMI of women of child-bearing age, which is measured as a continuous variable. Agricultural income (*AgInc*) is calculated as a product of total crop output produced by a household and household level sale prices of crop output. The function *f*(.) is an inverse hyperbolic sine (IHS), a transformation which works akin to a log transformation in terms of reducing the weight attached to extreme observations, but is defined for zero-valued observations [23]. Nevertheless, results are nearly identical when the transformation *g*(*AgInc*) = *ln*(*AgInc* + 1) is used, and are available upon request. Since changes in BMI are strongly correlated with changes in body weight, it is possible that shorter than one year time lags for explanatory variables, like agricultural income, may be appropriate for our analysis. For instance, it would be interesting to study seasonal effects of agricultural income by comparing women’s BMI following lean months of the agricultural season to BMI following harvest months. However, for such an analysis we would require higher frequency height/weight measurements, measured at least every season. In the absence of such data, we test for the association between agricultural income and BMI by averaging variation in agricultural income earned over the entire year to predict BMI. A one year lag in agricultural income also has the advantage of describing how agriculture income earned over the full agricultural cycle predicts nutritional status, rather than in the effects of income earned over a few months in the year. Earnings over the entire cycle are important because smallholders smooth consumption over the entire cycle and earnings decisions are correlated across seasons. *A*_*hvt*−1_ is total area in acres cultivated by a household in year *t* − 1, an important control-variable which intends to capture productivity differentials on account of land-size [24] which also likely correlate with nutritional differences across households and within individuals over time. We model participation in agriculture in a given year by including a participation dummy with *P*_*hvt*−1_ = 0 if *AgInc*_*hvt*−1_ = 0 and *P*_*hvt*−1_ = 1 if *AgInc*_*hvt*−1_ > 0. *α*_*ihv*_ is the constant and *ε*_*ihvt*_ is the mean zero error term.

The inclusion of individual level fixed effects (*γ*_*ih*_) differences out time-invariant individual-level factors which could potentially confound the effect of *AgInc*_*hvt*−1_ on *BMI*_*ihvt*_. Identification of the effect of *AgInc*_*hvt*−1_ on *BMI*_*ihvt*_ rests on the identifying assumption that time variant heterogeneity between individuals does not bias *β*_1_ on account of inducing correlation between *AgInc*_*hvt*−1_ and *ε*_*ihvt*_. Given our data, this assumption is not directly verifiable. Next best, we sequentially control for the most likely time-variant factors that could potentially account for the apparent relationship between *AgInc*_*hvt*−1_ and *BMI*_*ihvt*_ and do not find them to substantially alter our relationship of interest. In a separate specification Eq (2), described below, we also estimate the long term growth (over four years) of women’s BMI as a function of the growth of her household’s agricultural income, controlling for growth in other relevant dimensions, and find statistical support for our hypothesis. This specification, which averages out year-to-year fluctuations in BMI and Ag. Income, is less likely to be driven by idiosyncratic year-specific shocks. Taken together, both sets of results lend credibility to our identifying assumption.

We estimate the effect of agricultural income growth on BMI growth, over the long term as per the time-period of this study using:

$$g_{ihv(‘10-‘13)}^{BMI}=\alpha_{ihv}+\beta_{1}g_{hv(‘09-‘12)}^{AgInc}+\beta_{2}g_{hv(‘09-‘12)}^{A}+\beta_{3}g_{hv(‘09-‘12)}^{X}+\varepsilon_{ihv(‘10-‘13)}$$ (2)

Where, $g_{ihv}^{BMI}$ is the growth rate of women-specific BMI, $g_{hv}^{AgInc}$ is growth rate of household agricultural income, $g_{hv}^{A}$ is the growth rate of cultivated area and $g_{hv}^{X}$ is the growth rate of control variables. The growth rate of each included variable (measured at either the individual or household level) is calculated by estimating Eq (3) for every individual in the sample and capturing the coefficient on year (*t*). Eq (3) results from taking logs of the non-linear “exponential growth” equation *Y* = *α*(*e*)^*gt*^*ε*. Notice that this specification also implicitly accounts for an individual fixed-effect (for variables measured at the individual level) and household fixed-effect (for household level variables). Say, *c* denotes a fixed effect, and *Y* = *α*(*e*)^*gt*^ *εc*. Taking logs on both sides drops out the fixed effect (a dummy variable taking value of 1 for the relevant household) and we are back to estimating Eq (3).

$$ln\left(Y_{ih}\right)=ln\left(\alpha_{ih}\right)+g_{ih}t+ln\left(\varepsilon_{ih}\right)$$ (3)

Included time-variant controls (*X*_*ihvt*−1_) in Eq (1) and $g_{hv}^{X}$ in Eq (2) include changes in family size, changing access to amenities critical to both agricultural income and nutrition (household level access to electricity and piped water/water from a drinking water well), non-agricultural sources of household income, and village-level rainfall. One of the major strengths of the ICRISAT data is the detailed manner in which household income is tracked- not based on recall as is typical in surveys of this kind, but through monthly visitations to households. We include four major categories of non-agricultural income as controls- livestock income, income from non-agriculture (includes income from salaried jobs, income from caste occupations, business income, other non-farm income and income from non-farm migratory work), unearned income (includes gifts and remittances, savings and deposits and rental income) and income from agriculture labor. We also include village-level rainfall as a control in the model because it is both correlated with agricultural income and may also independently affect nutrition outcomes via altering the individual’s disease environment. A note of caution: despite a lagged specification of regressor variables and the inclusion of individual (and household) fixed effects as well as time-varying controls, it is important to keep in mind that potential sources of biases on account of unobserved time-variant heterogeneity may still be a source of bias for *AgInc*_*hvt*−1_ estimates and therefore the main findings of the paper are not accorded a causal interpretation.

An identification concern relevant in this context is one of reverse causality. This is the idea that better nourished individuals may be able to apply their labor more intensively in the agricultural production process and may hence enjoy higher output. Even though, clearly, BMI in our data is recorded after data on agricultural output for a year was collected, temporal persistence in BMI data could potentially invalidate our results. To check whether this is a concern in our context, we include lagged BMI (by a year) in our final specification as an explanatory variable. The inclusion of lagged BMI, has no effect on the estimated effect size of *AgInc*_*hvt*_, in fact the effect is somewhat strengthened. However, since anthropometric data collection started only in 2010, by including lagged BMI we lose all of our 2010 observations (BMI missing for 2009) and some additional observations for which BMI in the previous year was missing. Losing close to 40 percent of our observations nearly doubles our standard errors, making inference imprecise. These results have been omitted for brevity but are available upon request.

Finally, to account for unobserved aggregate shocks, we cluster our standard errors at the village level. Because we have a small number of villages (n = 18), we bootstrap standard errors on our coefficients of interest using Wild cluster bootstrapping [25]. This addresses concerns that with a small number of clusters standard asymptotic theory cannot be used to make inference and the use of standard distributional assumptions yield confidence intervals that are “too narrow”. To address concerns regarding serially correlated errors, we also alternatively cluster at the individual level, but in most cases these standard errors are smaller and therefore have been omitted for the sake of brevity, but are available upon request.

## Results

### Do agricultural incomes impact women’s nutritional status?

The extent to which the agricultural sector can influence individuals’ nutritional status, is a function of the size of the sector and its economic importance at the household-level. In the context of diversifying rural economic activity and the growing importance of the rural non-farm sector, the role of agriculture in poverty reduction and nutritional improvements, is not immediately obvious and requires detailed consideration. In S3 Fig we look at the sectoral composition of household incomes, to investigate the relative economic significance of farming activity. Income from farming (i.e. crop/agricultural income) is the largest source of income for households in our sample and, on average, accounts for around a third of all income. In comparison, the share of earned non-agricultural income, while on an upward trend, is still small, relative to farming. The “unearned income” category comprises of rental income (including rent from land), income from gifts and remittances and savings and deposits.

Thus, the break-down of the sectoral composition of household incomes posits an important role of agricultural incomes as a source of income for purchases and production of food for self-consumption. Agricultural income, vis-a-vis non-agricultural income, is a relatively more important source of income for women. Across both the non-agricultural sector and farming, a majority of income earned accrues to males. However, as can be seen in S4 Fig, the proportion of income accruing to women, is nearly two times as large in farming as it is in non-agriculture. This is consistent with time series data for India which suggests that the country is witnessing a feminization of the agricultural workforce as men shift rapidly to non agricultural sectors [26], [27]. To the extent that women spend more time working in farming than in non-agriculture, increases in agricultural output can plausibly afford women control over a larger share of household economic production and hence greater bargaining power over the allocation of household resources. The stated hypothesis is not tested in the current paper and evidence for the hypothesis in the South Asian context is inconclusive. However, the descriptive patterns highlighted here, in conjunction with the overall findings of the paper, motivate an explicit test of this hypothesis in the given context.

Table 1 presents results from our baseline specification (without secondary controls), of the effect of agricultural income on women’s BMI. Column (1) includes village fixed effects and column (2) includes individual fixed effects. Therefore, in column (1), we compare women’s BMI across households with differing agricultural incomes, within village and year. These estimates utilize cross-sectional variation across households and compared to the estimates in column (2) are demonstrably biased upwards. In column (2), we estimate individual-level deviations in BMI (from their person-specific means) as a function of year-wise deviations in household agricultural income and, hence, utilize within-individual variation in estimating the effect of agricultural incomes. These estimates are not confounded by observed and unobserved time-invariant differences between individuals that weaken the validity of cross-sectional estimates.

**Table 1:** Relationship between agricultural income & women’s BMI (Baseline Specification).

Independent Variable	Dependent Variable-BMI		
	(1)	(2)	(3)
Ag. Income	0.233**	0.102*/^+^	0.0791*/^+^
*(Cluster-Robust p-Value)*	*(0.033)*	*(0.085)*	*(0.082)*
*(Wild Bootstrap p-Value)*	*(0.034)*	*(0.106)*	*(0.108)*
Cultivated Area	0.0224	-0.00420	-0.00133
Ag. Sector Participation	-2.648**	-0.924	-0.694
Age	0.242***	-	-
Age Squared	-0.00207*	-	-
Constant	14.88***	20.01***	20.05***
Year FE	YES	YES	YES
Village FE	YES	YES	YES
Individual FE	NO	YES	YES
Extreme BMI Deviations Removed	NO	NO	YES
Observations	3,569	3,325	3,294

Notes: Standard errors are clustered at the village level. Variable for Ag. Income has been transformed using an inverse hyperbolic sine transformation. Cultivated Area is in acres, Ag. Sector Participation is a dummy variable for whether or not a household farms. Ag. Income, Cultivated Area, Ag. Sector Participation are measured for year (t-1) and BMI is for year t. “Age” and “Age-Squared” are important predictors of women’s BMI and are included in all cross-sectional/village fixed-effects specifications. Age variables are omitted from the panel/individual fixed-effects specifications because of the inclusion of year fixed-effects.

*** p<0.01,

** p<0.05,

* p<0.1,

^+^ p<0.15

In S2 Fig we see some individuals with very large BMI changes (<-4/>+4) over the time-period under consideration. Heights for these individuals were indeed recorded consistently, and large BMI changes are purely attributable to large changes in weight, a metric for which it is considerably harder to discern between actual changes versus measurement error. In column (3) of Table 1, we present estimates after excluding outliers. In particular, we exclude 15 smallest and 15 largest individual-level BMI deviations. In a subsequent section, we analyze the implications of our findings for the range of effect-sizes implied by our treatment of outlier observations. In Table 1 and in subsequent tables, for robustness, we present results first with no outliers dropped and next with 15 smallest and 15 largest observations dropped. To justify why we exclude 15 smallest and 15 largest BMI-deviations, in S2 Table we report how the effect-size changes when 5, 10 and 15 smallest and largest deviations are dropped from the sample. Dropping the 5 smallest and 5 largest deviations has the largest impact on our estimate of Ag. Income (point estimate drops by around 14.5 percent), but dropping subsequent observations have a much smaller impact on our point estimate of interest. Moreover, after dropping 15 smallest and 15 largest observations, the next 5 observations on either tail range from 10-14 kilos of weight change over the time period in our sample, which are plausible weight changes, especially considering that given that our panel is not a balanced one.

Table 2 presents results from the short term specification Eq (1) and sequentially adds in household-level controls (col. 1), village-level rainfall (col. 2), removes BMI deviation outliers (col. 3), and presents a final set of results with the full set of controls and without outliers (col. 4). Once we account for individual fixed effects, the effect of agricultural income on women’s BMI is robust to the inclusion of household level controls (compare estimates in column 2, Table 1 with column 1, Table 2). In column (2), we control for village-level annual rainfall, which only somewhat moderates the effect of *AgInc* on BMI. Dropping the 15 smallest and 15 largest BMI changes, results in a smaller but somewhat more precise effect-size (columns 3 & 4).

**Table 2:** Relationship between agricultural income and women’s BMI with sequential addition of controls (Panel-Data Results).

Independent Variable	Dependent Variable-BMI			
	(1)	(2)	(3)	(4)
Ag. Income	0.112*	0.103*	0.0887*	0.0809*
*(Cluster-Robust p-Value)*	*(0.056)*	*(0.071)*	*(0.052)*	*(0.057)*
*(Wild Bootstrap p-Value)*	*(0.074)*	*(0.088)*	*(0.064)*	*(0.066)*
Cultivated Area	-0.00292	-0.00583	0.0000639	-0.00251
Ag. Sector Participation	-1.017*	-0.931*	-0.784*	-0.707
Family Size	-0.0153	-0.0171	-0.0206	-0.0221
HH has Electricity	-0.182	-0.162	-0.214**	-0.194*
HH has Water	0.0666	0.0474	-0.00581	-0.0232
Livestock Income	-0.00591	-0.00576	-0.00235	-0.00224
Non- Ag. Income	0.000439	0.00226	0.00814	0.00987
Unearned Income	0.0242	0.0230	0.0132	0.0122
Ag. Labor Income	0.00998	0.0124	0.00937	0.0116
Constant	20.01***	19.68***	20.15***	19.85***
Year FE	YES	YES	YES	YES
Individual FE	YES	YES	YES	YES
Village Rainfall	NO	0.00425	NO	0.00391*
Extreme BMI Deviations Removed	NO	NO	YES	YES
Observations	3,325	3,325	3,294	3,294

Notes: Standard errors are clustered at the village level. All income variables have been transformed using an inverse hyperbolic sine transformation. All independent variables are lagged (t-1) and BMI is measured in year t.

*** p<0.01,

** p<0.05,

* p<0.1.,

+ p<0.15.

The sign of the coefficient on household level access to electricity (statistically significant in columns 3 and 4) is not in line with intuition. However, given high average levels of electrification in the sample villages (93-96 percent in 2010-2013), it is possible that households electrified during the time-period in our study, that is households electrified last, were the worst off households where no year-on-year improvements in BMI were seen. Nevertheless, as a first order concern, electricity at the household level is not correlated with household agricultural output, which is re-assuring. Notice that in all specifications the sign of the coefficient on agricultural sector participation is consistently large and negative. Given that we are utilizing within household variation, the effect is identified off the subset of households that farm in some years and not in others during the period of the study. The negative coefficient therefore indicates that, on average, years in which households farm, women of the household tend to have lower BMI relative to years in which the households do not farm. Without a complete understanding of a household’s decision to farm or not in a given year, interpreting this coefficient is difficult. It is possible that in years in which households farm, women exert more physical effort as compared to years in which they do not farm, which could partially explain the negative association between participation and BMI. However this interpretation is not causal because other factors that correlate with a households decision to farm in a given year and also simultaneously correlate negatively with BMI, are unobserved to us.

If instead of controlling for agricultural sector participation, we restrict our regression to only those household-year observations in which agricultural sector participation occurs (roughly 76 percent of the overall sample), our results are very similar to findings in Table 2.

To account for measurement error in BMI on account of some women being pregnant during the period of the study, we re-estimate Eq (1) by excluding woman-year observations during which a woman is pregnant. Results are reported in S3 Table. In line with expectation, a reduction in unexplained variation in BMI improves the precision of our estimates in some specifications. The effect size of *AgInc* also increases—the range of estimates for *AgInc* variable ranges from 0.106-0.131 points after removing pregnant observations, as opposed to 0.0809-0.112 points in Table 2.

One concern may be that the income variables included are not statistically significant and hence may not be serving as effective controls on account of being insufficiently correlated with BMI. In S4 Table, we re-estimate Table 2 with the four non-agricultural income variables being included as quartiles. As can be seen, higher quartiles of unearned income and agricultural labor income are indeed significantly correlated with better BMI outcomes. However, accounting for these controls does not substantially alter the effect of *AgInc*.

The linear-log relationship between BMI and household agricultural income, as modeled in Eq (1) implies a 10 percent increase in agricultural income is associated with a BMI increase of 0.008-0.0112 points, which is a 0.04-0.05 percent increase relative to mean BMI. To further give a sense of the economic implication of our results, we use the parameter-estimate obtained on the *AgInc* coefficient from different specifications of our individual fixed-effects model, to compare predicted BMI at specific levels of agricultural income. Using the parameter estimates from Table 2, column (2), we find that predicted BMI increases by 0.37 points when individuals between the 25th and the 75th percentile of the *AgInc* distribution are compared, with all other variables in the concerned regression, being held at their mean values. When parameter estimates from final specification Table 2 are used, we find that predicted BMI increases by 0.29 points, when individuals between the 25th and the 75th percentile of the *AgInc* distribution are compared. The difference in agricultural income in rupee terms between those at the 75th percentile of the transformed *AgInc* distribution and those at the 25th percentile, is of roughly 12,387 rupees (roughly 183 U.S. dollars or 710 PPP dollars) per acre per year. Averaged output prices across space and time imply that this rupee difference translates into yields of 0.43 tons/acre (pigeon pea) and 0.94 tons/acre (wheat).

Table 3 presents results from the long term growth specification in Eq (2). Column (1) includes zero valued year-by-household *AgInc* observations, and these results imply that a 10 percentage point increase in the growth rate of agricultural income is associated a 0.03 percentage point increase in the growth rate of BMI. Column (2) excludes zero-valued year-by-household *AgInc* observations. Around 90% of the excluded observations in column (2) are on account of households that don’t farm in all four years or in three out of four years. The effect of *AgInc* on BMI is stronger among these households, with a 10 percentage point increase in agricultural income growth rate being associated with a 0.10 percentage point increase in BMI growth rate. The somewhat counter intuitive negative association between the growth rate of livestock income and the growth rate of BMI requires consideration. On the one hand there exists a well documented association between animal husbandry and human diarrhea and enteric infections [28] which may plausibly explain the negative relationship. On the other hand livestock-raising is also considered to be a very labor intensive activity for women who predominantly care for livestock and collect fodder for livestock feed [29], [30]. For instance, authors in [31] document that more than 70% of the labor requirement for livestock production in India is provided by women. Both explanations are consistent with the patterns observed in the data and further research is necessary to understand the net nutritional implications of livestock raising for women. The growth rate of none of the other sources of non-agricultural income is statistically associated with the growth rate of BMI.

**Table 3:** Relationship between agricultural income growth and BMI growth.

Independent Variables	Dependent Variable-Growth Rate of BMI	
	(1)	(2)
	At least 2 years of AgInc ≥ 0	At least 2 years of AgInc > 0
Growth Rate of Ag. Income	0.00325***	0.0102***/**
*(Cluster-Robust p-Value)*	*(0.005)*	*(0.010)*
*(Wild Bootstrap p-Value)*	*(0.004)*	*(0.012)*
Growth rate of Cult. Area	-0.000542	-0.000621
Growth rate of Family Size	0.00175	0.0155
Growth rate of Water Access	-0.0148	-0.00460
Growth rate of Elec. Access	0.00346	0.0106
Growth rate of Non. Ag Income	-0.000832	-0.000487
Growth rate of Unearned Income	0.000369	0.000549
Growth rate of Livestock Income	-0.00298***	-0.00342**
Growth rate of Ag. Labor Income	0.000103	-0.000239
Growth rate of Rainfall	0.0261	0.0236
Constant	0.0175***	0.0159**
Observations	1,043	826

Notes: Standard errors are clustered at the village level. Growth rate for the independent variables is calculated for 2009-2012 and growth rate for BMI is calculated for 2010-2013.

*** p<0.01,

** p<0.05,

* p<0.1.

In S5 Table, we examine the effect of *AgInc* on women’s BMI, broken down by the age-category of the woman. Across all three specifications, the impact of agricultural income on BMI is driven by younger women in the age-group 15-25 years. The effect on younger women is also estimated very precisely. Therefore, the impact of agricultural income is stronger for women who are, on average, significantly more likely to be underweight (recall the age-wise distributions presented in S1B Fig). Alternative explanations may explain why diets of younger, more underweight women are more responsive to agriculture-income increases. An important literature in development economics suggests that households may follow a “pure investment strategy” in allocating intrahousehold resources during scarce times and cushion better endowed or more productive household members at the cost of more vulnerable household members [32], [33]. This, along with preference biases against younger women within the household may explain the finding. Both the “pure investment strategy” hypothesis and the “preference bias” hypothesis point to the low-status of younger women within the hierarchical structure of the Indian family. A well-established existence of son preference in India on account of several reasons–higher labor market returns to sons with whom parents typically live with in old age, the importance of sons for performing religious roles and rituals, and high dowry costs for daughters–designate a lower status to daughters within the Indian family [34]. Daughter-in-laws too suffer well established discrimination within the Indian household. For instance authors in [35] describe a layered hierarchy within which “older women in Indian families are subject to the authority of men, whereas supervision of younger daughters-in-law is delegated by men to older women”. Another separate explanation for the age-effect relates to the set point hypothesis whereby individuals have set points for their weight and stop eating once it is reached. For people at their set point (i.e. healthier individuals), an increase in agricultural income should not lead to an increase in their BMI.

### Empirical insights on agriculture-nutrition pathways

Next, we empirically test for the importance of home production for self-consumption, for nutrition. Under the non-separability of production and consumption decisions [36], which arise in the presence of high market-transaction costs, we might find home production of food to have a significant effect on nutrition.

In Table 4, we formally test for whether production for own consumption is a possible pathway by which agricultural incomes might impact women’s BMI. In an analogous regression, we examine the effects of food purchases, the results of which are presented in Table 5. We expect the two regressions to be symmetric, because own production and purchases together form well over 90% of the sourced by households (S5 Fig). S6 Fig shows overall expenditure shares for the different food groups among the sample households. In col. 1 of Tables 4 and 5 we test for the effect of the source of food procurement, by accounting for the ratio of expenditure on a food group from a certain source as a ratio of total expenditure on that food group, controlling for overall expenditure shares of included food-groups and total food expenditure. Col. 2 of both tables tests robustness to the exclusion of outlier BMI deviations.

**Table 4:** Own production of different food groups and women’s BMI.

Independent Variables	Dependent Variable-BMI	
	(1)	(2)
Own prod. ratio-cereals	0.230**/*	0.205*/^+^
*(Cluster-Robust p-Value)*	*(0.0494)*	*(0.0628)*
*(Wild Bootstrap p-Value)*	*(0.0760)*	*(0.140)*
Own prod. ratio-fruits & veg.	0.213	0.424
*(Cluster-Robust p-Value)*	*(0.747)*	*(0.426)*
*(Wild Bootstrap p-Value)*	*(0.908)*	*(0.616)*
Own prod. ratio-milk	0.0233	-0.0528
*(Cluster-Robust p-Value)*	*(0.887)*	*(0.636)*
*(Wild Bootstrap p-Value)*	*(0.887)*	*(0.640)*
Own prod. ratio-other foods	0.319	0.412
*(Cluster-Robust p-Value)*	*(0.791)*	*(0.668)*
*(Wild Bootstrap p-Value)*	*(0.890)*	*(0.730)*
Own prod. ratio-pulses	-0.389*	-0.260
*(Cluster-Robust p-Value)*	*(0.0716)*	*(0.142)*
*(Wild Bootstrap p-Value)*	*(0.0940)*	*(0.190)*
Overall expenditure share- cereals	1.861	0.842
Overall expenditure share- fruits & veg.	0.377	-0.539
Overall expenditure share- milk	3.232*	1.576
Overall expenditure share- other foods	2.174	1.784*
Overall expenditure share- pulses	0.760	-0.650
Total food expenditure	-0.178	-0.125
Constant	20.35***	20.68***
Year FE	YES	YES
Village FE	YES	YES
Individual FE	YES	YES
Extreme BMI Deviations Removed	NO	YES
Observations	3,325	3,294

Notes: Own production ratio for a food-group is the ratio of the imputed value (using market prices) of home production as a fraction of total expenditure on the item. Standard errors are clustered at the village level. Variable for “total food expenditure” has been transformed using an inverse hyperbolic sine transformation. All independent variables are lagged (t-1) and BMI is measured in year t.

*** p<0.01,

** p<0.05,

* p<0.1,

^+^ p<0.15.

**Table 5:** Food purchases of different food groups and women’s BMI.

Independent Variable	Dependent Variable-BMI	
	(1)	(2)
Purchase ratio-cereals	-0.244*	-0.227*/^+^
*(Cluster-Robust p-Value)*	*(0.0595)*	*(0.0580)*
*(Wild Bootstrap p-Value)*	*(0.0760)*	*(0.126)*
Purchase ratio-fruits & veg.	-0.276	-0.384
*(Cluster-Robust p-Value)*	*(0.620)*	*(0.406)*
*(Wild Bootstrap p-Value)*	*(0.844)*	*(0.580)*
Purchase ratio-milk	-0.0118	0.0277
*(Cluster-Robust p-Value)*	*(0.918)*	*(0.771)*
*(Wild Bootstrap p-Value)*	*(0.969)*	*(0.735)*
Purchase ratio-other foods	0.500	0.550
*(Cluster-Robust p-Value)*	*(0.210)*	*(0.146)*
*(Wild Bootstrap p-Value)*	*(0.168)*	*(0.112)*
Purchase ratio-pulses	0.514**/*	0.353*
*(Cluster-Robust p-Value)*	*(0.042)*	*(0.0680)*
*(Wild Bootstrap p-Value)*	*(0.066)*	*(0.086)*
Overall expenditure share- cereals	1.725	0.735
Overall expenditure share- fruits & veg.	0.157	-0.645
Overall expenditure share- milk	3.053*	1.436
Overall expenditure share- other foods	2.175	1.814*
Overall expenditure share- pulses	0.696	-0.596
Total food expenditure	-0.138	-0.0806
Year FE	YES	YES
Village FE	YES	YES
Individual FE	YES	YES
Extreme BMI Deviations Removed	NO	YES
Observations	3,325	3,294

Notes: Purchase ratio for a food-group is the ratio of the value of food purchase as a fraction of total expenditure on the item. Standard errors are clustered at the village level. Variable for “total food expenditure” has been transformed using an inverse hyperbolic sine transformation. All independent variables are lagged (t-1) and BMI is measured in year t.

*** p<0.01,

** p<0.05,

* p<0.1,

^+^ p<0.15.

Interesting patterns emerge when testing for the importance of own production versus purchase as pathways by which agricultural income might impact women’s BMI. Among the five food groups, only for cereals is production for own consumption statistically associated with increases in BMI, though this effect is somewhat imprecisely measured when outliers are removed (Table 4, col. 2). On the other hand, we see in Table 5 that the purchase of pulses has a relatively strong effect on women’s BMI. These findings are consistent with overall observations in the Indian context whereby farmers tend to sell more expensive, more nutritious produce on the market, retaining less lucrative cereals for self-consumption. For instance, authors in [37] who examine patterns of production, sale and consumption among farmers in the Vidarbha region of Maharashtra document that farmers sell 35-40% of Jowar and Wheat on the market, retaining 60-65% for household consumption and related purposes. In contrast, they find farmers to sell most pulses produced in the market at remunerative prices. In our results too, the negative coefficient on the own-production for consumption of pulses in Table 4 along with the positive and significant effect of pulse purchases in Table 5 indicates that Indian farming households do not set aside pulses they produce for consuming at home and instead purchase them from the market. Nutritional improvements on account of purchases of pulses among agricultural households, indicate that income increases may improve nutrition by enabling households to buy nutritious foods. Our overall findings suggest that income effects on account of agricultural earnings might be predominant for nutrition. While we do see that very large changes in non-agricultural income sources (yearly jump from the first to the fourth quartile) do correlate with BMI improvements, the short-term and long-term specifications taken together, suggest a dominant association of agricultural income and BMI.

## Concluding remarks

Agricultural productivity growth has long been seen as a promising pathway towards reducing malnutrition, given its high incidence among predominantly cultivator families in rural India. Our results encourage pursuing an agricultural growth strategy for addressing nutritional concerns, with a specific focus on women’s malnutrition. Moreover, our results suggest exceptionally stronger effects for younger women, a demographic most at risk of being underweight, and among whom fertility is largely concentrated. Among pathways considered, we find both own-production and market purchase of food to be associated with BMI increases but via different food groups. We find that while women’s BMI is positively associated with cereals produced and consumed at home, for more expensive and nutritious foods, the market plays an important role. Given the relative strength of rural markets in India, as compared to countries in sub-Saharan Africa, we provide an important context to evaluate the income-nutrition pathway via market access to food. That increasing agricultural incomes also empowers women within households to allocate expenses towards more nutritious purchases is a hypothesis that requires more detailed consideration, but is consistent with the patterns in our data.

Lastly, we also find a strong cross-sectional relationship between women’s BMI and that of her children, as measured by weight-for-height z-scores of children under 5 (S6 Table). Controlling for a set of village, household, mother and child level variables, mother’s BMI is a very strong predictor of her child’s weight-for-height z-score (p-value = 0.005). This effect exists net of differences in household socio-economics, and suggests a more direct link between maternal health with child weight. This effect is likely operational through multiple channels including, nutritionally, through breast-feeding, or more generally via mother’s caring capacity. This result, in conjunction with recent literature examining the effect of women’s empowerment on child nutrition [5], [38], suggest, additionally, strong inter-generational nutritional benefits of agricultural income increases.

## Supporting information

S1 Table Descriptive statistics of main variables used in statistical analysis. (PDF)

S2 Table Changes in effect-size of Ag. income due to removal of outliers. (PDF)

S3 Table Relationship between agricultural income and women’s BMI excluding pregnant woman-years (Panel-Data Results). (PDF)

S4 Table Relationship between agricultural income and women’s BMI with income quartiles as controls. (PDF)

S5 Table Relationship between agricultural income and women’s BMI- by Age. (PDF)

S6 Table Relationship between child’s weight-for-height & mother’s BMI. (PDF)

S1 Fig Cross-sectional BMI distribution of sample women. (TIF)

S2 Fig Individual-level BMI variation of sample women. (TIF)

S3 Fig Sectoral composition of household incomes by year. (TIF)

S4 Fig Proportion of income accruing to women and men from non-ag. sector & farming. (TIF)

S5 Fig Sources of food procurement by year and food group. (TIF)

S6 Fig Household-level food group expenditure share by year. (TIF)
