# Supplementary material for: The role of agriculture in women’s nutrition: Empirical evidence from India
Source: PLoS One. 2018 Aug 15;13(8):e0201115. doi: 10.1371/journal.pone.0201115 (PMC6093637; doi:10.1371/journal.pone.0201115)
Supplement: S1 Table — (PDF) [file pone.0201115.s001.pdf]

Table S1: Descriptive Statistics of Main Variables used in Statistical Analysis

	2009		2010		2011		2012		2013	
	Mean	SD	Mean	SD	Mean	SD	Mean	SD	Mean	SD
BMI	-	-	20.44	3.71	20.47	3.75	20.45	3.64	20.7	3.67
Age (in years)	29.9	9.87	30.54	10.03	30.5	9.85	30.46	9.91	30.71	10.06
Education (in years)	5.71	4.96	5.71	4.95	5.96	4.96	6.23	5.01	6.37	5
Household Size (no. of people)	6.12	2.84	5.95	2.8	5.96	2.8	6	2.83	6.12	2.92
Cultivated Area (in acres)	5.6	8.91	5.82	9.02	6.42	11.17	6.14	10.65	6.45	11
% HHs in Farming	0.76	0.42	0.76	0.42	0.78	0.42	0.78	0.42	0.81	0.4
Ag. Income ('000 rupees)	88.19	201.79	94.17	161.08	86.08	147.68	96.38	170.44	110.51	208.42
Livestock Income ('000 rupees)	31.54	42	34.26	43.77	39.25	53.93	46.1	61.51	39.22	57.18
Non-Ag. Income ('000 rupees)	24.2	57.58	30.7	65.18	37.43	71.4	39.78	71.74	38.9	67.89
Unearned Income ('000 rupees)	33.28	86.93	48.36	236.86	39.37	94.14	38.6	80.23	34.74	86.66
Ag. Labor Income ('000 rupees)	11.74	16.22	13.68	17.86	14.26	20.71	12.69	18.12	12.06	16.97
Total HH Income ('000 rupees)	189	263.23	221.52	343.33	216.8	227.36	233.91	237.41	235.64	274.45
Medical Expenditure ('000 rupees)	4.84	11.38	5.15	14.07	4.4	8.79	5.29	11.08	4.96	10.21
HH Food Expenditure ('000 rupees)	35.63	17.59	35.38	17.54	37.47	19.36	37.51	19.5	38.37	20.74
Cereal Share in Food Expenditure	0.25	0.08	0.22	0.06	0.22	0.07	0.22	0.08	0.2	0.08
% HHs with electricity	0.88	0.33	0.93	0.26	0.94	0.24	0.96	0.21	0.96	0.19
% HHs with water	0.52	0.5	0.51	0.5	0.53	0.5	0.53	0.5	0.56	0.5
% HHs with toilets	0.25	0.43	0.28	0.45	0.35	0.48	0.37	0.48	0.42	0.49
Village rainfall (cm/year)	78.76	17.27	100.3	21.82	74.58	26.16	61.11	25.18	90.81	29.49

Notes: All income & expenditure variables are in real terms, expressed in 2009-10 rupees.
