# Supplementary material for: The role of agriculture in women’s nutrition: Empirical evidence from India
Source: PLoS One. 2018 Aug 15;13(8):e0201115. doi: 10.1371/journal.pone.0201115 (PMC6093637; doi:10.1371/journal.pone.0201115)
Supplement: S2 Table — (PDF) [file pone.0201115.s002.pdf]

Table S2: Changes in Effect-Size of Ag. Income due to Removal of Outliers

Independent Variable	Dependent Variable-BMI			
	None	5 smallest & 5 largest	10 smallest & 10 largest	15 smallest & 15 largest
Ag. Income	0.102*/$^{+}$	0.0872*	0.0833*/$^{+}$	0.0791*/$^{+}$
(Cluster-Robust p-Value)	*(0.085)*	*(0.077)*	*(0.083)*	*(0.082)*
(Wild Bootstrap p-Value)	*(0.106)*	*(0.096)*	*(0.108)*	*(0.108)*
Cultivated Area	-0.00420	0.000483	-0.00125	-0.00133
Ag. Sector Participation	-0.924	-0.752	-0.692	-0.694
Constant	20.01***	20.00***	20.00***	20.05***
Year FE	YES	YES	YES	YES
Village FE	YES	YES	YES	YES
Individual FE	YES	YES	YES	YES
Observations	3,325	3,314	3,305	3,294

Notes: Standard errors are clustered at the village level. *** p<0.01, ** p<0.05, * p<0.1., + p<0.15
