# Supplementary material for: The role of agriculture in women’s nutrition: Empirical evidence from India
Source: PLoS One. 2018 Aug 15;13(8):e0201115. doi: 10.1371/journal.pone.0201115 (PMC6093637; doi:10.1371/journal.pone.0201115)
Supplement: S3 Table — (PDF) [file pone.0201115.s003.pdf]

Table S3: Relationship between Agricultural Income and Women's BMI excluding Pregnant Woman-Years (Panel-Data Results)

Independent Variable	Dependent Variable-BMI			
	(1)	(2)	(3)	(4)
Ag. Income	0.131**	0.125**	0.113*	0.106*
(Cluster-Robust p-Value)	*(0.045)*	*(0.047)*	*(0.057)*	*(0.056)*
(Wild Bootstrap p-Value)	*(0.054)*	*(0.048)*	*(0.086)*	*(0.076)*
Cultivated Area	-0.00823	-0.0111	-0.00433	-0.00704
Ag. Sector Participation	-1.283*	-1.218*	-1.069*	-1.009*
Family Size	-0.0131	-0.0142	0.00893	0.00779
HH has Electricity	-0.249**	-0.229**	-0.224**	-0.205*
HH has Water	0.0110	-0.00701	-0.0309	-0.0476
Livestock Income	-0.0101	-0.00992	-0.00320	-0.00300
Non- Ag. Income	-0.000143	0.00146	0.00386	0.00538
Unearned Income	0.0185	0.0175	0.00819	0.00732
Ag. Labor Income	0.00603	0.00814	0.00482	0.00679
Constant	20.25***	19.96***	20.12***	19.84***
Year FE	YES	YES	YES	YES
Individual FE	YES	YES	YES	YES
Village Rainfall	NO	0.00377	NO	0.00354
Extreme BMI Deviations Removed	NO	NO	YES	YES
Observations	3,083	3,083	3,074	3,074

Notes: Standard errors are clustered at the village level. *** p<0.01, ** p<0.05, * p<0.1., + p<0.15
