# Supplementary material for: The role of agriculture in women’s nutrition: Empirical evidence from India
Source: PLoS One. 2018 Aug 15;13(8):e0201115. doi: 10.1371/journal.pone.0201115 (PMC6093637; doi:10.1371/journal.pone.0201115)
Supplement: S4 Table — (PDF) [file pone.0201115.s004.pdf]

Table S4: Relationship between Agricultural Income and Women's BMI with Income Quartiles as Controls (Panel-Data Results)

Independent Variable	Dependent Variable-BMI			
	(1)	(2)	(3)	(4)
Ag. Income	0.105*	0.0962*	0.0873*	0.0794*
(Cluster-Robust p-Value)	*(0.081)*	*(0.100)*	*(0.074)*	*(0.092)*
(Wild Bootstrap p-Value)	*(0.098)*	*(0.112)*	*(0.084)*	*(0.096)*
Cultivated Area	-0.00349	-0.00664	0.00139	-0.00143
Ag. Sector Participation	-0.942	-0.854	-0.742	-0.664
Family Size	-0.0208	-0.0235	-0.0126	-0.0150
HH has Electricity	-0.157	-0.135	-0.207*	-0.186*
HH has Water	0.0467	0.0268	0.0141	-0.00347
2nd Quartile Non-Ag. Income	-0.0333	-0.0216	-0.110	-0.0990
3rd Quartile Non-Ag. Income	-0.0829	-0.0627	-0.00538	0.0126
4th Quartile Non-Ag. Income	0.0849	0.115	0.170	0.197
2nd Quartile Livestock Income	-0.0636	-0.0528	0.00595	0.0156
3rd Quartile Livestock Income	-0.0218	-0.0342	0.0500	0.0387
4th Quartile Livestock Income	-0.0919	-0.115	-0.00193	-0.0229
2nd Quartile Unearned Income	0.114	0.126	0.0447	0.0558
3rd Quartile Unearned Income	0.00848	0.0136	-0.0442	-0.0396
4th Quartile Unearned Income	0.177*	0.195**	0.139	0.156*
2nd Quartile Ag. Labor Income	0.0554	0.0827	-0.000132	0.0243
3rd Quartile Ag. Labor Income	0.130	0.150	0.100	0.118
4th Quartile Ag. Labor Income	0.0781	0.113	0.186*	0.216**
Constant	20.16***	19.79***	20.11***	19.78***
Year FE	YES	YES	YES	YES
Individual FE	YES	YES	YES	YES
Village Rainfall	NO	0.00469*	NO	0.00419*
Extreme BMI Deviations Removed	NO	NO	YES	YES
Observations	3,325	3,325	3,314	3,314

Notes: Standard errors are clustered at the village level. *** p<0.01, ** p<0.05, * p<0.1, + p<0.15.
