# Supplementary material for: The role of agriculture in women’s nutrition: Empirical evidence from India
Source: PLoS One. 2018 Aug 15;13(8):e0201115. doi: 10.1371/journal.pone.0201115 (PMC6093637; doi:10.1371/journal.pone.0201115)
Supplement: S5 Table — (PDF) [file pone.0201115.s005.pdf]

Table S5: Relationship between Agricultural Income and Women's BMI- by Age

Independent Variable	Dependent Variable-BMI		
	(1)	(2)	(3)
Younger#Ag. Income	0.131**	0.121**	0.0999**
(Cluster-Robust p-Value)	*(0.021)*	*(0.024)*	*(0.015)*
(Wild Bootstrap p-Value)	*(0.046)*	*(0.048)*	*(0.044)*
Older#Ag. Income	0.101^{+}	0.0928^{+}	0.0703^{+}
(Cluster-Robust p-Value)	*(0.106)*	*(0.131)*	*(0.131)*
(Wild Bootstrap p-Value)	*(0.142)*	*(0.154)*	*(0.158)*
Age Group	0.203	0.225	0.0918
Cultivated Area	-0.00242	-0.00531	-0.00210
Ag. Sector Participation	-0.958	-0.876	-0.654
Family Size	-0.00590	-0.00761	-0.0148
HH has Electricity	-0.185*	-0.164	-0.197*
HH has Water	0.0722	0.0533	-0.0178
Livestock Income	-0.00591	-0.00575	-0.00223
Non- Ag. Income	-8.72e-05	0.00173	0.00942
Unearned Income	0.0236	0.0224	0.0118
Ag. Labor Income	0.0102	0.0125	0.0117
Village Rainfall	NO	0.00424	0.00388*
Constant	19.84***	19.50***	19.73***
Year FE	YES	YES	YES
Individual FE	YES	YES	YES
Extreme BMI Deviations Removed	NO	NO	YES
Observations	3,325	3,325	3,294

Notes: Standard errors are clustered at the village level. Age Group=0 if $25 < \text{age} \leq 49$ and Age Group=1 if $15 \leq \text{age} \leq 25$. *** p<0.01, ** p<0.05, * p<0.1, + p< 0.15.
