# Supplementary material for: The role of agriculture in women’s nutrition: Empirical evidence from India
Source: PLoS One. 2018 Aug 15;13(8):e0201115. doi: 10.1371/journal.pone.0201115 (PMC6093637; doi:10.1371/journal.pone.0201115)
Supplement: S6 Table — (PDF) [file pone.0201115.s006.pdf]

Table S6: Relationship between Child's Weight-for-Height & Mother's BMI

Independent Variable	Dependent Variable- WHZ	
Mother's BMI	0.0622***	0.0656***
(Cluster-Robust p-Value)	*(0.005)*	*(0.004)*
(Wild Bootstrap p-Value)	*(0.004)*	*(0.004)*
Age in Months	-0.00726	-0.00724
Sex	-0.106	-0.113
Birth Order	-0.0163	-0.0123
Mother's Education	0.00979	0.00915
Mother's Age	-0.0149	-0.0177
Ag. Income	0.0601	0.0494
Cultivated Area	-0.00768	-0.00612
Ag. Sector Participation	-0.295	-0.184
Livestock Income	0.00778	0.00696
Non- Ag. Income	0.0239*	0.0228*
Unearned Income	0.0457***	0.0455***
Ag. Labor Income	-0.00112	-0.00308
Constant	-1.981**	-1.286*
HH has Water	0.129	0.107
HH has Toilet	0.0439	0.0297
Village Rainfall	NO	-0.00819
Observations	709	709

Notes: Standard errors are clustered at the village level. All income variables have been transformed using an inverse hyperbolic sine transformation. *** $p<0.01$, ** $p<0.05$, * $p<0.1$, + $p<0.15$.
