# Supplementary figures and images for: The role of agriculture in women’s nutrition: Empirical evidence from India
Source: PLoS One. 2018 Aug 15;13(8):e0201115. doi: 10.1371/journal.pone.0201115 (PMC6093637; doi:10.1371/journal.pone.0201115)

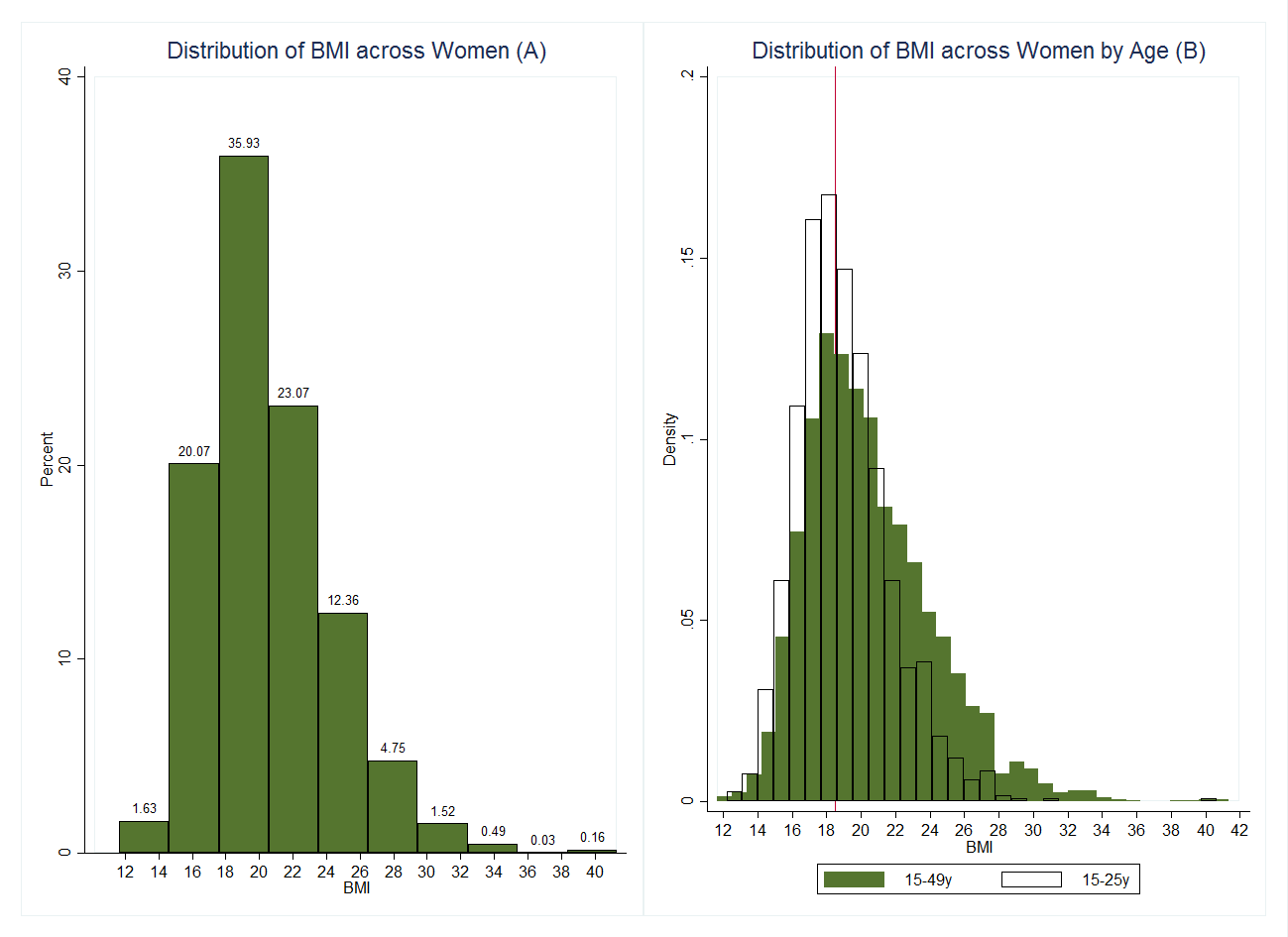

Supplement: S1 Fig — (TIF) [file pone.0201115.s007.tif]

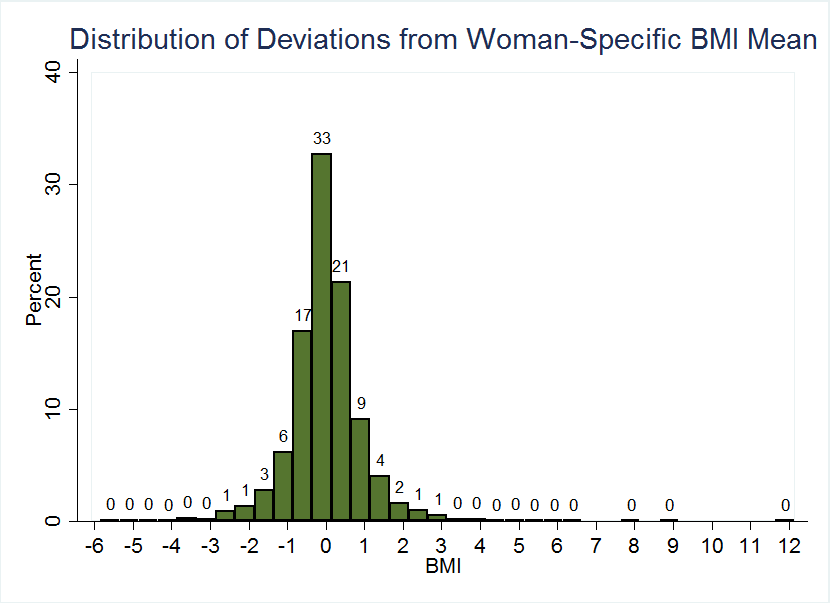

Supplement: S2 Fig — (TIF) [file pone.0201115.s008.tif]

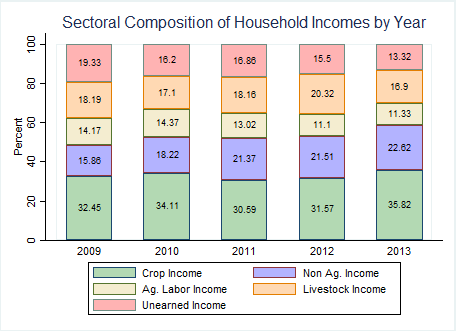

Supplement: S3 Fig — (TIF) [file pone.0201115.s009.tif]

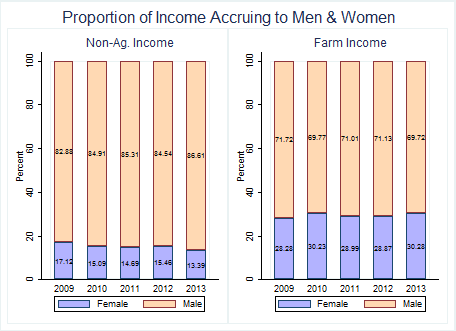

Supplement: S4 Fig — (TIF) [file pone.0201115.s010.tif]

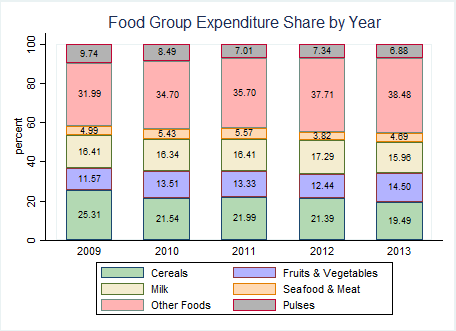

Supplement: S5 Fig — (TIF) [file pone.0201115.s011.tif]

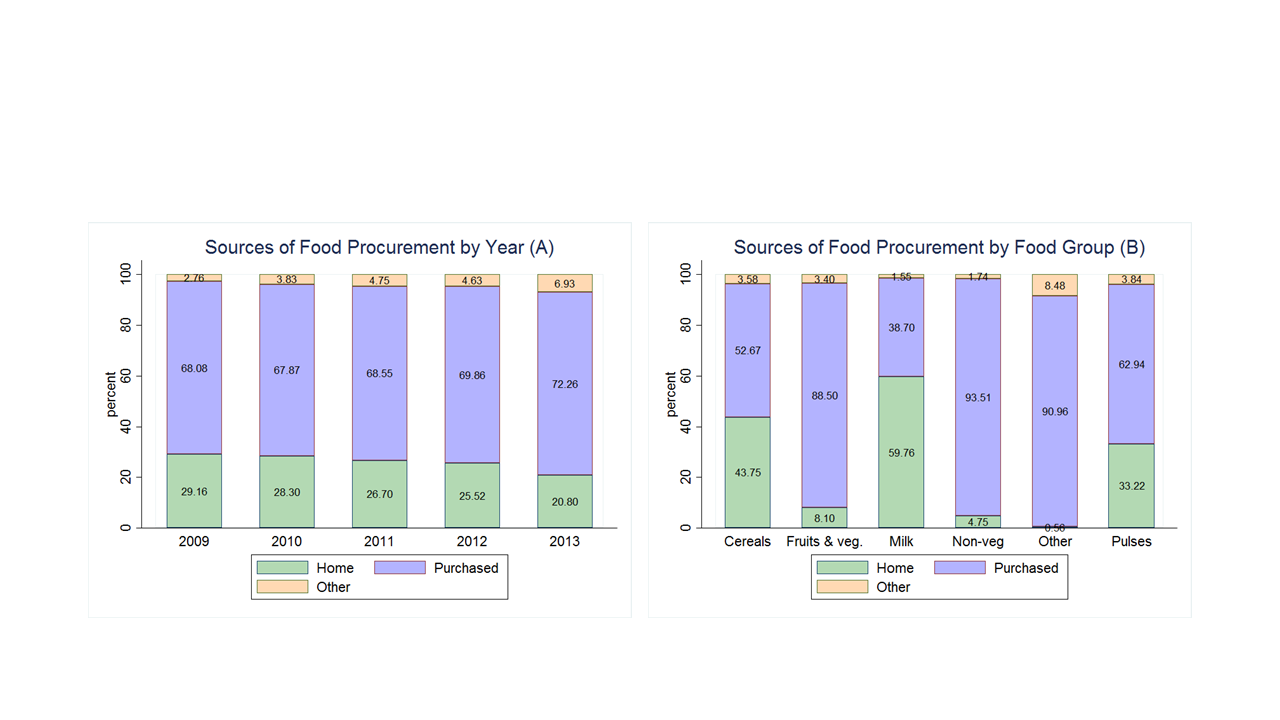

Supplement: S6 Fig — (TIF) [file pone.0201115.s012.tif]
